# Supplementary material for: GLN: a method to reveal unique properties of lasso type topology in proteins
Source: Sci Rep. 2020 Sep 16;10:15186. doi: 10.1038/s41598-020-71874-2 (PMC7494857; doi:10.1038/s41598-020-71874-2)
Supplement: Supplementary file 1 — Supplementary material 1 [file 41598_2020_71874_MOESM1_ESM.pdf]

GLN – a method to reveal unique properties of lasso type topology in proteins

Supplementary Material

Wanda Niemyska, Kenneth C. Millett and Joanna I. Sulkowska

Contents

1 The Gauss integral for two closed curves and $max|GLN|$ for protein with disulfide bridge. 1

2 Dataset. 3

3 Examples of "the same" GLN matrices for proteins with different folds. 4

4 Classification of loops in proteins. Comparison of the GLN technique and the minimal surface technique. 5

5 Intriguing cases having different classifications in the GLN technique and the minimal surface technique. 7

6 Application of the GLN method – reaction coordinates to study the non-trivial folding and unfolding pathway. 8

1 The Gauss integral for two closed curves and $max|GLN|$ for protein with disulfide bridge.

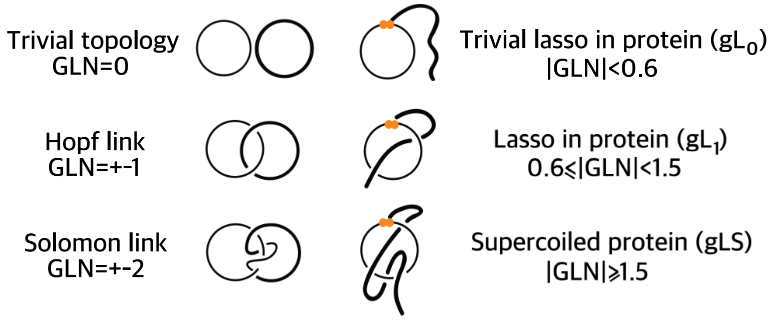

Figure S1: Two left columns: examples of the values of the Gauss integral for two closed curves, with schematic pictures of the simplest links. Two right columns: examples of the corresponding $|whGLN|$ values based on proteins with a disulfide bridge (one closed loop) and one terminus (second open loop), with schematic pictures of the basic lasso types.

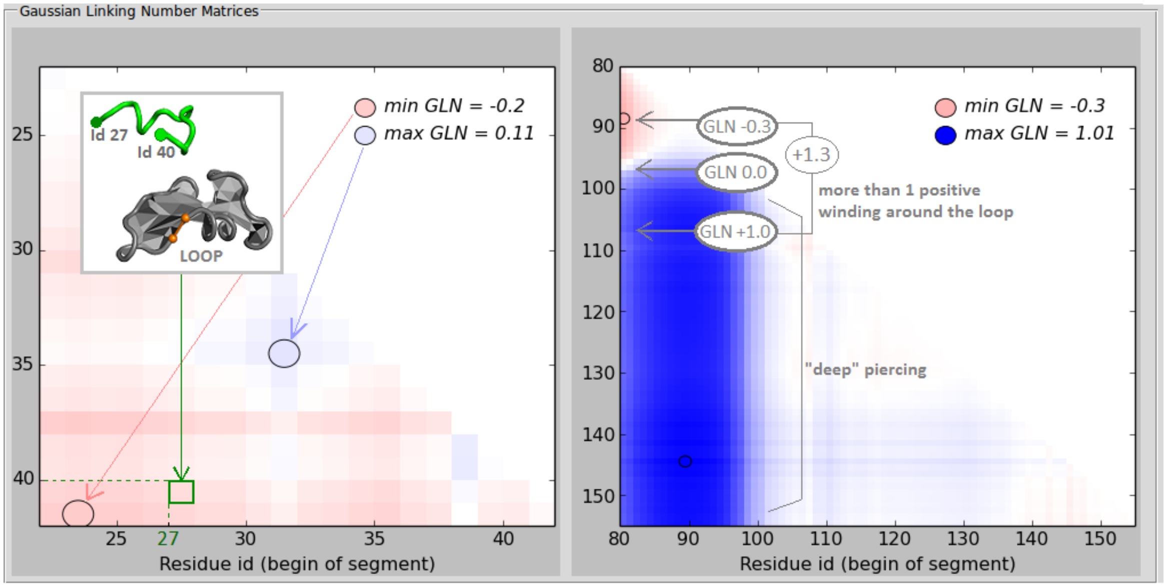

Figure S2: Examples showing how to interpret elements of the GLN matrices – the topological fingerprints. Each cell of the matrix corresponds to the specific subchain of the tail, where the id of the first residue is on the x-axis and the id of the last residue is on the y-axis. Thus the left bottom corner corresponds to the whole tail. The color intensity indicates the value of the GLN, red shows negative values connected with windings in negative direction, while blue shows positive windings. Left panel: the structure of gL_0 type, with no significant windings. Subchains for which minimum and maximum GLN values are achieved are indiucated on the matrix (subchains from id 23 to id 41, and from id 31 to id 34, respectively). The diagram of the loop (with gray surface spanned on it) and fragment of the tail from id 27 to id 40 is inserted. It is easy to see that this fragment does not wind around the loop and thus corresponding area on the matrix is bright. Right panel: the structure of gL_1 type, with one large clear blue patch indicating that tail winds around the loop in positive direction. Following downward on the left edge of the matrix, important observations can be made. In this case the beginning of the analyzed segment remains the same - beginning of the tail - while the end of the analyzed segment is moving toward the end of the tail. Between residue ids 88 and 107, GLN value increases by 1.3, which means that this fragment of the tail fully encircules the loop and crosses it somewhere. The blue color that does not change along the rest of left edge indicates that long tail remained does not wind fruther around the loop and stays on the same side of the loop. The figure is made with *PyLasso* plugin [1].

2 Dataset.

We used the set of 5,106 non-redundant proteins with at least one bridge from LassoProt database [2], March 2016. By *non-redundant* we mean the sequence similarity lower than 35%, including X-ray, NMR, CEM structures, and proteins with unresolved parts. We chose only one chain from each protein and have identified 13,320 covalent loops in a total.

Among 5,106 proteins in the dataset, there were 1,276 structures with unresolved parts. These were reconstructed using Gaprepairer [3] based on Modeller [4]. In the modeling procedure, missing fragments were reconstructed based on the homological structure. In case a homological structure is not known and the number of missing atoms is smaller than 10 amino acids, we used the Modeller loop prediction method. In this set (also called a set of artifacts), 26 structures were classified as error chains in LassoProt database. This means that the distances between Cα atoms are wrong, i.e., out of the range 2.0-4.2 A, or that the indices of residues are in the wrong order. We identified in a total 4,051 covalent loops in artifacts or error chains. In some analyses, we considered only the remaining set of 9,269 loops. In some analyses, we sparately considered each pair of a loop and one tail. Since there are 1,520 loops having just one tail (with the second one of length 0 or 1) in our set of non-artifact chains, we considered 17,018 structures.

3 Examples of "the same" GLN matrices for proteins with different folds.

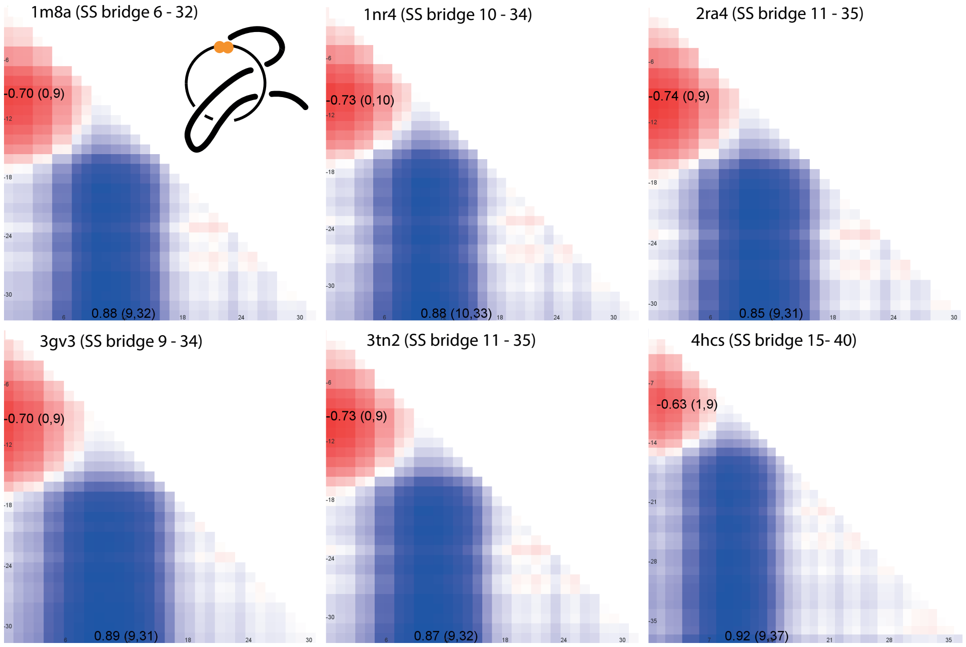

Figure S3: Examples of the GLN matrices for proteins with very low sequence similarity and a different fold but the same GLN motif gL_2 (for at least one loop; the scheme of motif gL_2 is given in the top left corner). The PDB ids of proteins and ids of the residues which close loops are printed on the top of each matrix. A description of the GLN matrix is shown in Fig S2.

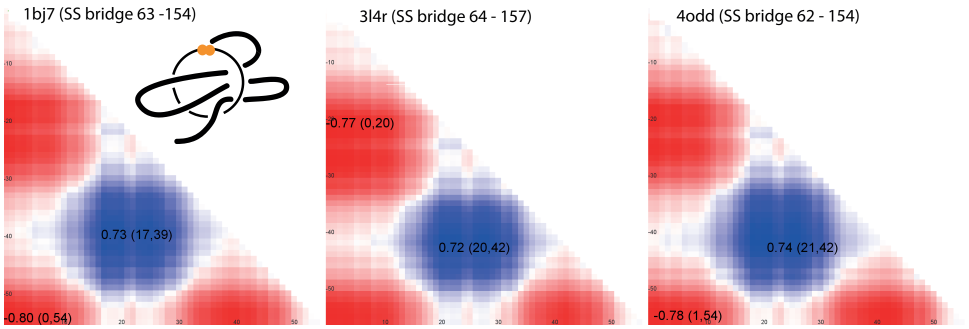

Figure S4: Additional examples of the GLN matrices for proteins with very low sequence similarity and a different fold but having the same GLN motif, this time gL_3. The description of the matrices the same as in Fig S3.

4 Classification of loops in proteins. Comparison of the GLN technique and the minimal surface technique.

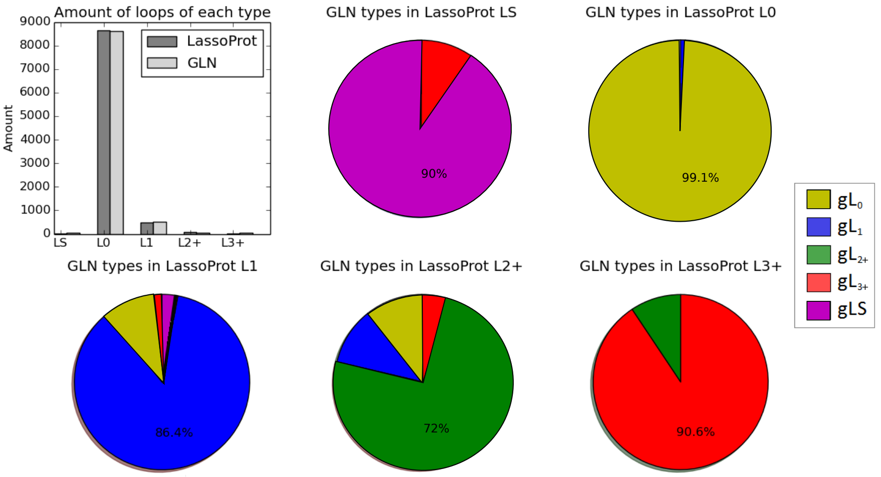

Figure S5: Classification of loops in proteins. In the LassoProt database [2; 5], lassos are classified using the minimal surface method. When the following constants are used $tr_1 = 0.69, tr_2 = 1.5, tr_3 = 0.55$ in the lasso definition based on GLN, then as many as 98% of loops are classified in analogous way by both techniques. The biggest coincidence is among loops of lasso type L_0, where over 99% of them are of type gL_0. Around 90% of loops of type LS or L_{3+} are classified as gLS and gL_{3+}, respectively, while the lowest coincidence occurs in the case of type L_{2+} - where only 72% of the loops of this type are classified as gL_{2+}.

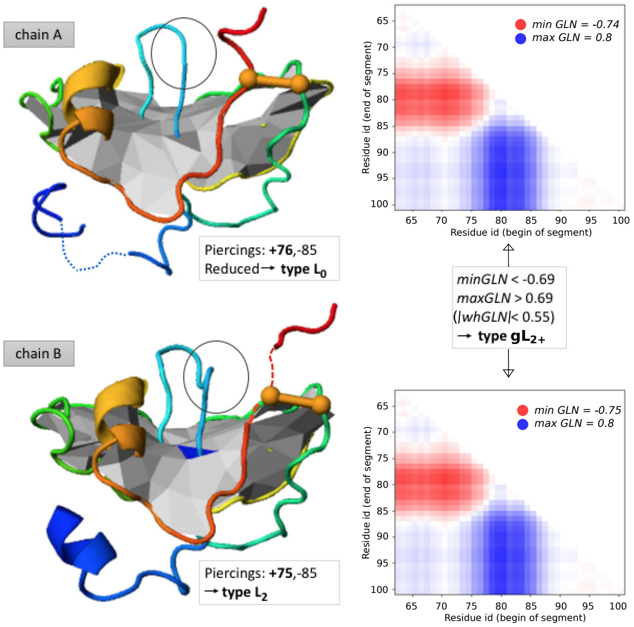

Figure S6: An example of the difference in the classification between the minimal surface and GLN technique. On the left: cartoon representations of the domains of protein (PDB id 3nsw), chains A and B, with cysteine bridges between residue ids 3 and 62 (in orange), and minimal surfaces spanned by the loops (in gray). In the inset we give the ids of residues that cross the surface (sign indicates direction of piercing). On the right: the GLN matrices corresponing to C-tails of both chains. The loop in chain A is classified as of L_0 type by the minimal surface technique, while the loop in chain B is classified as of L_2 type. This is due to the fact that two segments of chain A that cross the minimal surface spanned on the loop are close enough (namely, there is less than 10 amino acids between them) to be reduced by the algorithm, as it considers them insignificant or artificial. The GLN technique classifies both structures as of gL_{2+} type (the inset in right column shows the rules used to classify structures as gL_{2+}. Both structures satisfy them). Perhaps, both techniques might be best combined to give the most robust classification scheme.

5 Intriguing cases having different classifications in the GLN technique and the minimal surface technique.

Table S1: Intriguing cases with different classifications using the GLN technique and the minimal surface technique. These are loops from non-redundant chains, they are not artefacts and have minimal surfaces spanned on them that are pierced, but they have low values of $max|GLN|$ that are not greater than 0.6. We found only 9 such loops (0.01% in the analyzed dataset). In the column $Max|GLN|$ minus indicates that maximum $|GLN|$ is achieved for negative GLN value. In the column *Piercings* we provide the ids of the residues that cross minimal surfaces spanning the loops. The signs indicate the direction of crossings. Notice that the maximal distance between the loop and piercings in this dataset is 16 residues, while the average one is lower than 9. This suggests that the piercings are quite shallow (as is suggested by lower GLN value), but not shallow enough to reduce them using the minimal surface technique.

**Protein (chain)**	**Loop range**	**Tail**	**Piercings *(distance from loop)***	**Max|GLN|**
3jdl (A)	28–540	N	-24 *(4)*	(-) 0.5
1ypy (A)	49–136	C	+149, -159 *(13)*	(-) 0.54
2yhg (A)	564–779	N	+557 *(13)*	0.54
3qsd (A)	133–199	N	-127 *(6)*	(-) 0.55
1nyo (A)	8–142	C	-153 *(11)*	(-) 0.56
4yfc (B)	31–126	C	+129 *(3)*	0.57
1gp0 (A)	1,598–1,634	C	+1,641 *(7)*	0.58
4i05 (A)	133–199	N	-127 *(6)*	(-) 0.6
1le6 (A)	48–122	N	-32 *(16)*	(-) 0.6

6 Application of the GLN method – reaction coordinates to study the non-trivial folding and unfolding pathway.

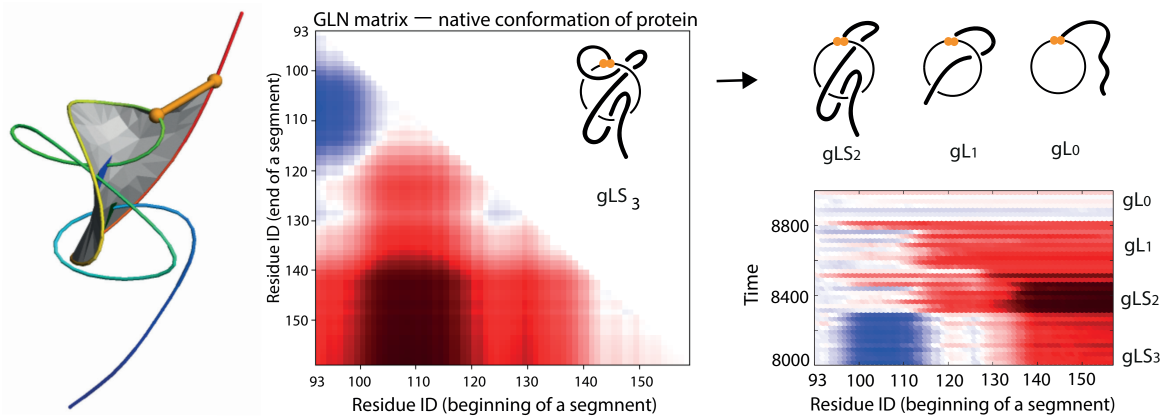

Figure S7: Analysis of the unfolding pathway of the MANEC-type domain from the hepatocyte growth factor activator inhibitor-1 (PDB ID 2msx, loop composed by residues 50–92). This protein has a gLS_3 motif (called supercoiling), where the C-terminal tail winds around the loop and crosses it two times sequentially from the same site. Left panel: smoothed cartoon representation of the protein chain, with the cysteine bridge in orange and the surface in gray. Middle panel: the GLN matrix of the native conformation. Right panel: visualization of one of the possible unfolding routes for the resulting lasso protein derived from a structure-based coarse-grained model with molecular dynamics simulation. The matrix shows the unfolding route via a slipknot topology $gLS_3 \rightarrow gLS_2 \rightarrow gL_1 \rightarrow gL_0$. Each row of this matrix corresponds to a single time frame of the simulation and represents the left edge of the GLN matrix for this frame.

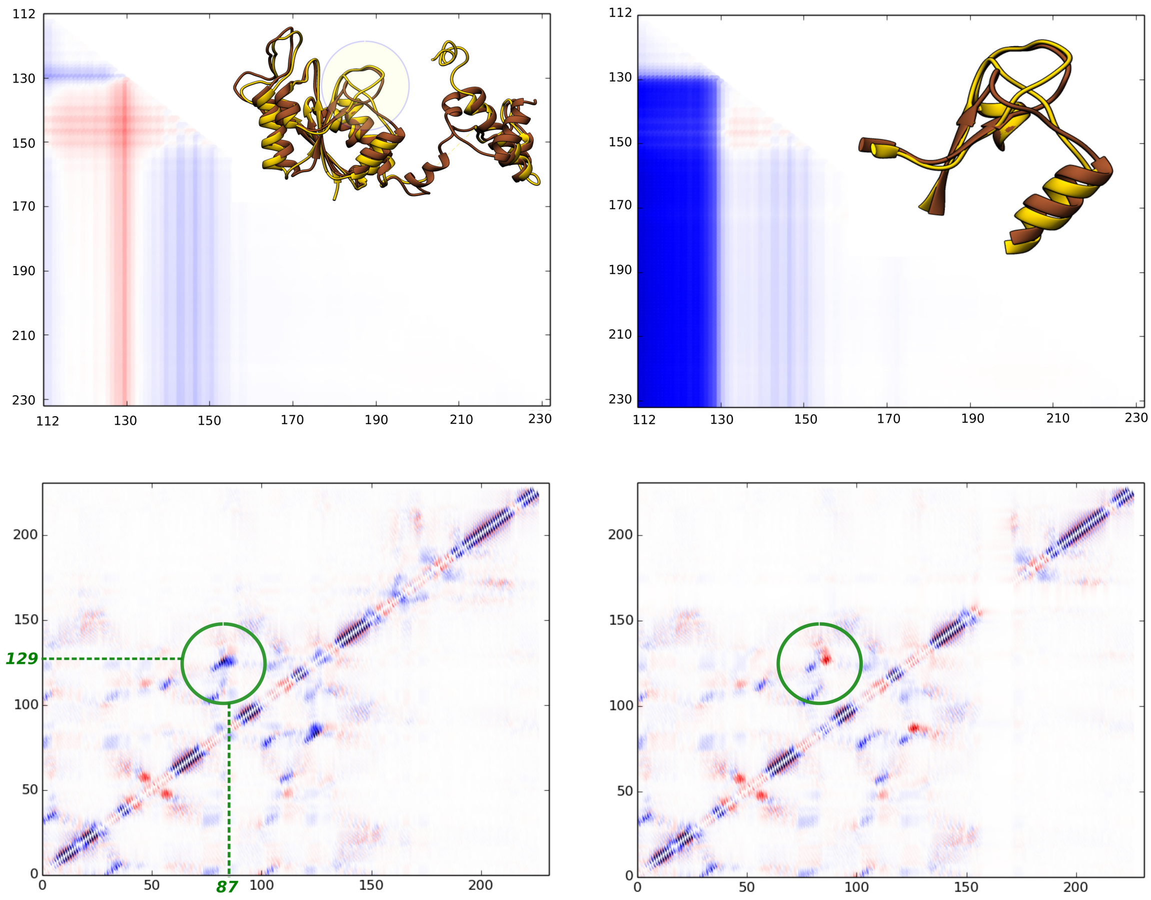

Figure S8: Comparison of the GLN matrices for knotted (left) and unknotted (right) conformations of the same protein, PDB ID 1oy5. This protein was crystalized as unknotted, however, it is expected that it is knotted since it was shown that the knotted topology is strictly conserved for homologous proteins [6]. The knotted homological protein (PDB ID 4yqd) was used to model knotted version of 1oy5. On the left there are matrices for the original structure with the unresolved part and, on the right, the structure that has its unresolved part modelled. The insets focus on the knot and unknot locations. Top row: the "standard" GLN matrices, for the "loop" comprised of residues ids 86–111 and the C terminal tail. In the left inset, cartoon representations of oryginal structure (in brown) and the modeled one (in yellow) are superimposed, with the circle fragment of chain forming the loop (86–111) indicated. In the right inset, the crucial fragment is cut out and enlarged. In the original structure, the brown fragment of the tail (around residue id 130) passes nearby the brown loop outside of it and not forming a knot. The modeled yellow fragment of tail passes nearby the yellow loop, crossing it on inside and, in this way, forming a trefoil knot (3_1). This case demonstrates how the GLN matrices show motifs gL_0 and gL_1, respectively. Bottom row: in these matrices the GLN values between all pairs of unit segments of protein chain are presented. Without colors, they would look the same and would barely differ from contact maps (compare Fig. S9). The place in the matrices which is colored differently (marked by green circle) corresponds to the GLN between residue ids, approximately, 87 and 129. The first piece is in the loop, while the second is passing nearby the loop - on the outside and the inside for the original and the modeled structures, respectively. They show the changes of direction of entanglement between those pieces, i.e. the sign of GLN value and the color on the matrix. Notice that even without prior knowledge of the shape of those structures, i.e. if one doesn't know the presence of the "loop" 86–111, we could suspect the difference in topology by comparing these matrices. This demonstrates why the GLN fingerprint may serve as reference value for a reaction coordinate with which one can study folding pathways of protein with complex topology.

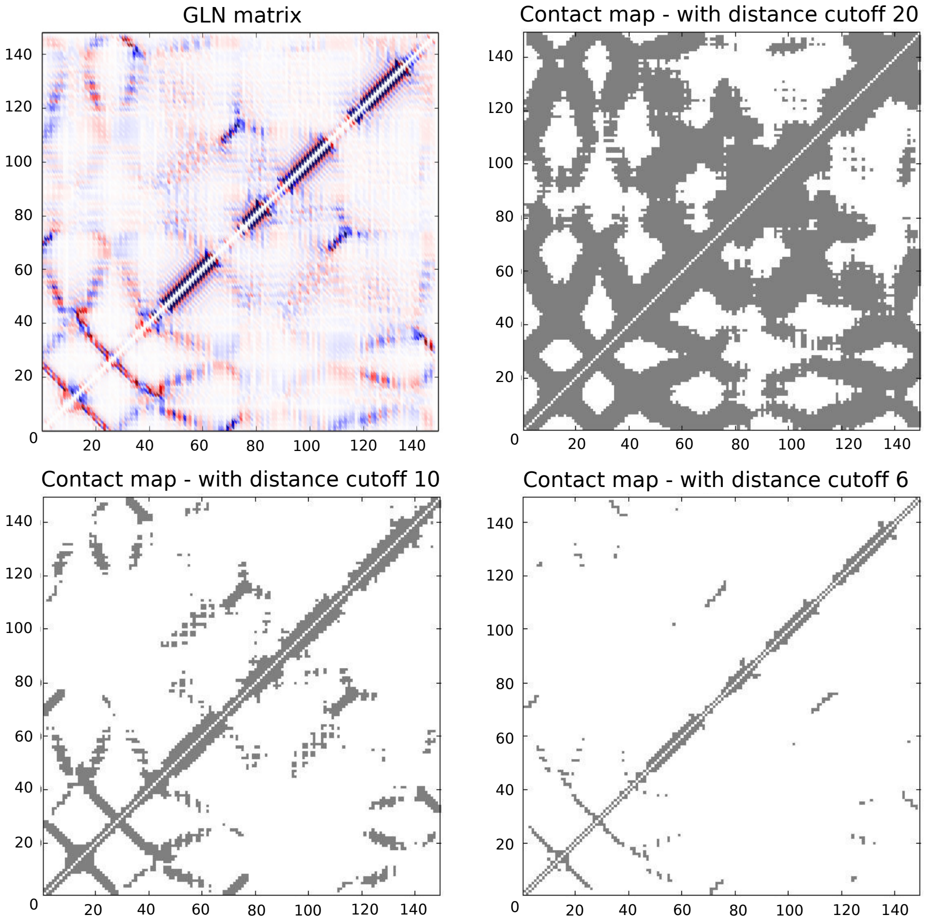

Figure S9: GLN matrix (with GLN values between all pairs of segments) and three contact maps with different distance cutoffs for hydrolase, pdb ID 2ehg. All of them reveal the same pattern - this is because the GLN value strongly depends on distance. Thus the GLN fingerprint can also be used to identify type of secondary structures in protein which are usually visible via a contact map. Note, the shape of the contact map depends on the cutoff distance used to determine physical contacts while GLN does not depend on additional parameters. Moreover, the sign of the GLN (blue or red color on the matrix) indicates the "direction of contact", i.e. from this it can be deduced on which side the fragments of the protein chain are in contact and pass each other. Thus the GLN fingerprint of the native conformation can be used as a reference value for a reaction coordinate with which to study folding pathways of protein, especially for those with complex topology.

References

[1] A. M. Gierut, W. Niemyska, P. Dabrowski-Tumanski, P. Sułkowski, J. I. Sulkowska, Pylasso: a pymol plugin to identify lassos, Bioinformatics 33 (23) (2017) 3819–3821.

[2] P. Dabrowski-Tumanski, W. Niemyska, P. Pasznik, J. I. Sulkowska, Lassoprot: server to analyze biopolymers with lassos, Nucleic acids research 44 (W1) (2016) W383–W389.

[3] A. I. Jarmolinska, M. Kadlof, P. Dabrowski-Tumanski, J. I. Sulkowska, Gaprepairer: a server to model a structural gap and validate it using topological analysis, Bioinformatics 34 (19) (2018) 3300–3307.

[4] B. Webb, A. Sali, Protein structure modeling with modeller, Protein Structure Prediction (2014) 1–15.

[5] W. Niemyska, P. Dabrowski-Tumanski, M. Kadlof, E. Haglund, P. Sułkowski, J. I. Sulkowska, Complex lasso: new entangled motifs in proteins, Scientific reports 6 (2016) 36895.

[6] J. I. Sułkowska, E. J. Rawdon, K. C. Millett, J. N. Onuchic, A. Stasiak, Conservation of complex knotting and slipknotting patterns in proteins, Proceedings of the National Academy of Sciences 109 (26) (2012) E1715–E1723.
